# Editorial: The role of foliar nutrition and biostimulants in increasing crop adaptation to environmental stresses, volume II

**DOI:** 10.3389/fpls.2023.1234731

**Published:** 2023-08-01

**Authors:** Ebrahim Hadavi, Jose M. Garcia-Mina

**Affiliations:** ^1^ Independent Researcher, Avignon, France; ^2^ Department of Chemistry, University of Navarra, Pamplona, Spain

**Keywords:** foliar nutrition, foliar biostimulation, plant stress alleviation, abiotic and biotic stress, sustainable agriculture

In recent years, the utilization of foliar nutrition and biostimulants as effective strategies to enhance crop adaptation to environmental stresses has attracted significant attention. A wealth of research, including literature reviews, has explored the applications of these interventions as foliar sprays, shedding light on their promising benefits.

One crucial study that consolidates the findings from various sources is a comprehensive review conducted by Bhupenchandra et al.. This review evaluated the effectiveness of different foliar biostimulants, such as humic substances and seaweed extracts, across multiple crops. The researchers synthesized the findings from various studies and highlighted the positive outcomes associated with foliar biostimulant applications. The review revealed consistent improvements in root development, nutrient uptake, and drought tolerance in response to foliar biostimulants. This comprehensive analysis underscores the versatility and efficacy of foliar sprays in promoting crop adaptation to environmental stresses.

Additionally, Jacquens et al. investigated the effects of foliar nutrient applications on wheat crops under heat stress conditions. The study demonstrated that foliar sprays containing specific nutrients, such as calcium and potassium, significantly mitigated the adverse effects of heat stress on wheat plants. The application of these nutrients as foliar sprays resulted in improved physiological processes, including reduced oxidative damage, enhanced photosynthesis, and increased yield stability. These findings highlight the potential of foliar nutrition in safeguarding crop performance in the face of challenging environmental conditions.

Furthermore, the other studies have contributed valuable insights into the potential of foliar nutrition and biostimulants as foliar sprays. Kandil et al.⁠ focused on rice cultivation and demonstrated the significance of addressing specific nutrient deficiencies through foliar zinc applications. The researchers observed significant increases in rice grain yield and improved nutrient uptake efficiency. This study highlights the targeted delivery of nutrients through foliar sprays as an effective means of enhancing crop adaptation.

Similarly, Abd El-Mageed et al.⁠ investigated the coapplication of effective microorganisms (EMs) and nanomagnesium as foliar sprays on sweet potato plants. Their findings showcased the considerable benefits of this intervention, including enhanced growth, improved photosynthesis, and increased stress tolerance. By delivering biostimulants directly to the plant through foliar sprays, this study reinforces the potential of these applications in enhancing crop adaptation to environmental stresses.

Moreover, Bello et al.⁠ explored the effects of a microalgae extract, applied as a foliar spray, on bell pepper plants subjected to drought stress. Their research revealed significant improvements in plant growth, photosynthetic efficiency, and antioxidant activity. This study provides further evidence for the efficacy of foliar biostimulants in promoting crop adaptation under specific stress conditions.

When considering the wider context, tailoring interventions to meet the specific needs of different crops emerges as a critical aspect of optimizing crop performance. Harish et al. investigated into the benefits of zero-tillage and foliar phosphorus (P) nutrition in a maize-wheat rotation system. Their findings emphasized the importance of crop-specific approaches, as the combination of these interventions improved nutrient availability and soil water conservation, ultimately enhancing overall productivity.

The collective findings consistently underscore the efficacy of foliar sprays in enhancing crop adaptation to environmental stresses. By harnessing the potential of foliar nutrition and biostimulants, farmers and agronomists can employ targeted strategies to address nutrient deficiencies, enhance growth, and increase stress tolerance in crops. Additionally, the versatility of foliar sprays allows for the effective delivery of biostimulants, facilitating crop-specific responses to stressors.

In conclusion, the studies reviewed in this editorial, along with the comprehensive analysis conducted by Bhupenchandra et al., provide substantial evidence supporting the role of foliar nutrition and biostimulants as foliar sprays in increasing crop adaptation to environmental stresses. The foliar application is an alternative method to other resource demanding methods like soil application and it is expected to continue gaining more popularity due to growing economic and environmental concerns in the contemporary world and especially in undeveloped and developing countries. By leveraging the benefits of these interventions, agriculture can move towards sustainable practices, ensuring optimized crop performance and enhanced resilience in the face of evolving environmental challenges.

## Author contributions

EH authored the manuscript, and JG-M reviewed it. All authors contributed to the article and approved the submitted version.

